# What it takes to build a health services innovation training program

**DOI:** 10.5116/ijme.61af.30bd

**Published:** 2021-12-23

**Authors:** Elizabeth Martin, Megan Campbell, William Parsonage, David Rosengren, Scott C. Bell, Nick Graves

**Affiliations:** 1 Australian Centre for Health Services Innovation, Centre for Healthcare Transformation, School of Public Health and Social Work, Queensland University of Technology, Australia; 2 Royal Brisbane and Women's Hospital, Australia; 3 Translational Research Institute, University of Queensland, Australia; 4 Health Services and Systems Research, SingHealth Duke-NUS Health Services Research Institute, Singapore

**Keywords:** Health services innovation, Capacity building, Implementation science

## Introduction

Health services need to constantly innovate for value and efficiency.^1^^,^^2^ Globally, there is increased demand for healthcare because of changing population profiles and the rapid development of health technologies.^3^^,^^4^ At the same time, health services and governments attempt to slow or stop increases in health expenditure.^5^ Together, these challenges create an environment where choices inevitably need to be made about which models of healthcare to deliver and how best to deliver them. Sound innovations are required throughout the entire health system, solving a broad range of problems, improving value for patients and efficiency for health systems.

There is value in medical education that drives health service improvement. Such education needs to reach all innovative clinicians. Clinicians can span the boundary between research and practice because they have a deep understanding of the health service context, can develop a strong multidisciplinary research network, and can advocate within the health service for support.^6^ Clinician innovators can often gain traction with traditional doctors and are respected and trusted by other clinicians, senior managers and politicians. They carry more political influence, conferred by high public trust.^7^^-^^9^ Translating, mobilizing and applying research knowledge to address practical health service challenges and make informed decisions about the allocation of scarce resources is therefore more successful.^10^^,^^11^

Skills in health services innovation, including implementation science, understanding health systems and economic evaluation, are vital because they span the innovation journey from ideation to evaluation but are rarely part of medical education. In response, health services often look to workplace training programs to build staff capacity for innovation, focussing on specific skills like project management, leadership and evaluation. However, this type of medical education is often only a few days long, not supported over the long term,^12^ and participants are likely to experience inconsistency with language and techniques across multiple programs.^13^^-^^16^ A short course cannot confer the skills needed to complete and publish good quality health services research, and therefore many health innovations may never be disseminated. Only a few programs give participants a university qualification that they can leverage for health services research, higher degree qualifications or career progression. Elements of health services innovation are likely to be taught in the six implementation science courses offered by universities internationally,^17^^-^^22^ and health economics and health management courses are widely available. However, we are not aware of many university courses that combine cost-effectiveness and implementation science to build health services capacity for innovation.^23^

The purpose of this paper is to describe the co-design and implementation of a post-graduate course and broader program of support in health services innovation and how a partnership between a university and the largest health service in Australia facilitated the program. We aim to share our experiences and methods to inform other university-health service partners of a potential approach for developing clinician innovators through medical education and building health services innovation capacity.

### The implementation science approach

The Consolidated Framework for Implementation Research (CFIR)^24^ was used to structure our description of the medical education intervention development and implementation. The CFIR was chosen because the process required engagement with individuals and groups across multiple levels of the health service and also external stakeholders. The CFIR provided the best framework to describe how the stakeholders interacted and led the educational intervention.^25^ The intervention is hereafter referred to as the 'the program'. We first describe key elements of the inner and outer settings for the program's development, with characteristics of key individuals embedded into these CFIR constructs. We then describe the program components and the process of co-design and implementation. These constructs enable essential program elements to be organized and adapted by other university-health service partnerships.

### Inner Setting

The partner health service is one of the largest in Australia, servicing a population of 900 000 people, has an annual budget of AUD 3 billion and five hospitals with inpatient and ambulatory capabilities, two of which are tertiary/quaternary hospitals. It delivers a broad range of community and public health services and some state-wide services.^26^^,^^27^ The health service had a strongly supported research strategy,^28^ with a new and vigorous focus on value-based healthcare, providing an excellent climate for program implementation. There was a strong engagement of the executive leadership team, and a significant proportion of the research strategy resources were allocated to the program. The established executive-level partnerships with the university, particularly around health services research, were vital to the program's genesis.

Queensland University of Technology (QUT) led the program implementation via its established Australian Centre for Health Services Innovation.^29^ The Centre was established in 2011, partnering with other universities and health services to build capacity for health services research, drive knowledge translation through training and skills development and address the frustratingly slow pace of adoption of innovation amongst Australian health services.^29^ Academics saw the challenges experienced by health services as untenable, and therefore there was large tension for change and partnership. Two of the core ways the Centre achieved improved implementation of innovation was through academic clinician partnerships and increased training of clinicians in health services innovation.

The health service was a founding partner of the Centre, and in 2016 asked the Centre to configure several short courses into a university award course, which became the Graduate Certificate in Health Science (Health Services Innovation). Key health service executive directors were champions for the short courses in implementation science and cost-effectiveness analysis offered by the Centre. Their support and good working relationship with the Centre directors was the catalyst for tailoring the short courses and transforming them into a post-graduate university program that supported innovation within the health service. The program was a natural extension of the University Centre's aims. Academics in the Centre are willing to take risks,^30^ and therefore amongst the program leadership and operational staff, there was a large absorptive capacity for developing and implementing the program, especially as it was mostly funded by the health service. By June 2017, both partners were committed to the decision to develop and implement the program.

The program was continuously championed by these key health service executive directors who participated in promotional videos. Strong support was also received by the university executive dean who had committed attendance at orientation and graduation celebrations and other university and health service directors who stepped up to provide governance during leadership changes. The first cohort of students was selected by the health service because of their seniority and influence in the health service. This group of health service staff were initially not familiar with the program and not sure why they were 'tapped on the shoulder' but were willing to engage as 'early adopters'. The first two groups of students in particular, were skilled, enthusiastic and committed to sustained use of knowledge and new capabilities. They were also proud to be involved in the health service leading something innovative and new. Feedback was openly received from this cohort, and the students were reassured by the university staff's willingness to listen and adapt. All individuals from both organizations demonstrated great commitment to continuous improvement and innovation from an educational perspective, underpinned by a strong self-belief in their capability to execute the program.

### Outer Setting

There were key aspects of the outer setting that facilitated the program within the health service. As with most health services nationally, the health needs of the community are complex and diverse. The health service is well networked to multiple universities across the city, and as one of the state's largest and commensurately highest funded health services, research is both expected and supported.^28^ There is also large pressure from health service funders, national and state governments to reduce the costs of care. Top-up funding ceased in 2017, and growth in funding was capped in 2015 despite growing community needs.^31^^,^^32^ These pressures have created a strong motivation for innovation that improves the value of care. This motivation and culture is strengthened nationally by several government and non-government agencies' commitment to value-based healthcare which is driving health services innovation through a desire to lower costs and improve health outcomes.^33^^-^^35^

### Educational intervention

These facilitating elements of the inner and outer organizational contexts accelerated university-health service discussions about how to encourage health services innovation and improve the value of healthcare. The course was co-designed, with dedicated university and health service staff working collaboratively on curriculum development and strategic executive-level guidance provided by a course advisory group. There was no available evidence that delivering a university

post-graduate course would be an effective intervention for driving health services innovation and value, yet the health service adopted the university Centre's risk-taking approach to innovation and acknowledged that evidence justifying the inclusion of individual elements warranted the development of the program overall. There are three core components of the program: delivery of post-graduate level units (subjects), student support services and innovation implementation support.

The four units comprising the academic curriculum were designed to deliver content in the prescribed order so that students could slowly build their confidence to plan, implement and evaluate new ideas or improvements to the health service. The four units are: Implementation Science: Theory and Application in Health; Health Systems; Cost-Effectiveness Analysis for Healthcare Decision Making; and an independent workplace-based project. One unit is completed per semester, with face-to-face lectures delivered on the university campus in block format over consecutive days. The aim of creating exclusive cohorts of students and bringing them together face to face is to facilitate internal relationship-building and peer support networks within the health service. Students usually complete the academic curriculum in two years.

Student support services and innovation implementation support is led by a university academic who streamlines the university experience for students, providing a single point of contact, triaging, redirecting or solving issues as they arise. Consequently, the academic develops close relationships with the students and is capable of matching students with university supervisors for completion of the workplace-based project. The health service also appointed a dedicated professional to the program. The purpose of this role is to facilitate the strategic support and alignment of students' study and workplace-based projects within the health service. The additional two elements of support provided to students – both administrative and project-focused across the two organizations – were included in the program as essential elements that ensured the application of the academic curriculum and continued engagement of students who were busy clinicians and managers who have extreme competing demands, including during a pandemic.

Critical elements of the program were the benefits seen by both partners, its adaptability and the health service's ability to mitigate the complexity of implementing the program within its large health service. The program is charged on a cost-recovery basis by the university, which in lieu of profit, provides opportunities for the university-based researchers to engage with clinicians and pursue long-term research relationships. The university also gains an advantage through its growing reputation of delivering a bespoke and adaptable course. As with most innovations, adaptation was necessary. One example of adaptation is that as subsequent cohorts commence the course, there is less of an assumption that students are confident with embarking on the post-graduate study, have good academic writing skills and have experience with research. Another example of adaptation was minor changes to assessment based on feedback, ensuring the tasks remain relevant to the health service. Adaptation is not difficult for the health service or university as there are sufficient governance processes in place for receiving feedback, generating, and approving ideas. The program is, however, complex for the health service, engaging multiple campuses, disciplines and 'middle management'. Middle managers are difficult to engage and/or are largely overlooked in healthcare innovation and require a 'road' or organizational structure that connects them to both innovation practice and executive support for innovation.^36^^,^^37^ We realized this after using the CFIR^24^ for ongoing reflection and formal evaluation, which commenced after the first year. The program is marketed internally within the health service as one that promotes excellence but also 'you belong here and are welcome'. Marketing and messaging are comprehensive and widespread, but within an organization of 17 000 staff,^38^ it is difficult to reach all staff, especially middle managers. The characteristics of the educational intervention, particularly the credible genesis of the intervention, the relative advantages it poses to both the health service and the university and its adaptability are unique aspects that require careful governance and implementation processes.

### Process

The program is governed by a course advisory group with members from the health service, university and external experts to provide independent advice. The group was formed in 2017 to plan the course structure and additional support program. Operational staff across both organizations developed the detailed curriculum and position descriptions for the university and health service roles that provided the additional support program. No specific implementation framework was adopted for planning. However, staff who governed and operationalized the program were experienced academics and clinicians who were intuitively aware of what it would take to plan a program that was a sustained success.

Another important early stage was the engagement of health service executives by both the sponsor and director of the program. In-principle support was received from the chief executive officer of the health service, and the program was formally approved by all executive directors of the health service. Executive directors were then asked to identify and engage 'early adopters'; staff who had capacity, confidence, reach and influence for selection as the first cohort of the program. The engagement of these early and critical stakeholders resulted in a set of approximately 45 champions across the health service, which built momentum for the program in subsequent years.

## Conclusion

We have used the CFIR to describe the co-design and implementation of a medical, educational intervention that aims to build clinician innovators' capacity and capability and their health service. This approach highlights the complex program requirements such as strong, existing relationships, willingness to take risks and the adaptability of the intervention. We recommend similar university-health service partnerships address the enduring efficiency challenges experienced by the health sector and use an implementation science framework to examine how best to build health service innovation capacity.

### Conflict of Interest

The authors declare that they have no conflict of interest.
